# Fatal attraction in glycolysis: how *Saccharomyces cerevisiae *manages sudden transitions to high glucose

**DOI:** 10.15698/mic2014.01.133

**Published:** 2014-02-20

**Authors:** Johan H. v. Heerden, Meike T. Wortel, Frank J. Bruggeman, Joseph J. Heijnen, Yves J. Bollen, Robert Planqué, Josephus Hulshof, Tom G. O’Toole, S. A. Wahl, Bas Teusink

**Affiliations:** 1 Systems Bioinformatics/AIMMS/NISB, VU University, De Boelelaan 1085, 1081HV Amsterdam, The Netherlands.; 2 Department of Biotechnology, Delft University of Technology, Julianalaan 67, Delft 2628 BC, Netherlands.; 3 Kluyver Centre for Genomics of Industrial Fermentation/NCSB, Delft University of Technology, Julianalaan 67, Delft 2628 BC, The Netherlands.; 4 Department of Molecular Cell Biology, VU University, De Boelelaan 1085, 1081HV Amsterdam, The Netherlands.; 5 LaserLaB Amsterdam, VU University, De Boelelaan 1085, 1081HV Amsterdam, The Netherlands.; 6 Department of Mathematics, VU University, De Boelelaan 1085, 1081HV Amsterdam, The Netherlands.; 7 Department of Molecular Cell Biology and Immunology, Vrije University Medical Center, v/d Boechorststraat 7, 1081 BT Amsterdam, The Netherlands.

**Keywords:** carbon metabolism, glycolysis, dynamic regulation, metabolic model, bistability, heterogeneity, yeast

## Abstract

In the model eukaryote *Saccharomyces cerevisiae*, it has long been known that a functional trehalose pathway is indispensable for transitions to high glucose conditions. Upon addition of glucose, cells with a defect in trehalose 6-phosphate synthase (*Tps1*), the first committed step in the trehalose pathway, display what we have termed an *imbalanced glycolytic state; *in this state the flux through the upper part of glycolysis outpaces that through the lower part of glycolysis. As a consequence, the intermediate fructose 1,6-bisphosphate (FBP) accumulates at low concentrations of ATP and inorganic phosphate (P_i_). Despite significant research efforts, a satisfactory understanding of the regulatory role that trehalose metabolism plays during such transitions has remained infamously unresolved. In a recent study, we demonstrate that the startup of glycolysis exhibits *two* dynamic fates: a proper, functional, steady state or the imbalanced state described above. Both states are stable, attracting states, and the probability distribution of initial states determines the fate of a yeast cell exposed to glucose. Trehalose metabolism steers the dynamics of glycolysis towards the proper functional state through its ATP hydrolysis activity; a mechanism that ensures that the demand and supply of ATP is balanced with P_i_ availability under dynamic conditions. [van Heerden *et al.* Science (2014), DOI: 10.1126/science.1245114.]

The general design of glycolysis, with an ATP consuming upper-part and an ATP producing lower-part, comes with major risk if sudden increases in flux are not managed properly. When excess glucose becomes available, its rapid phosphorylation results in the decrease of ATP and P_i_ as glycolysis is initiated. In response, regulatory mechanisms are required to ensure that ATP production by lower glycolysis can keep up with ATP consumption by the upper-part; failure to do so results in a dysfunctional state, which we call an imbalanced glycolytic state, and loss of cell viability. The consequences of this failure have been extensively studied in S. *cerevisiae*, where the trehalose pathway has been implicated as a key component of the regulatory machinery that ensures robust transitions to high glucose.

*S. cerevisiae* *tps1*Δ mutants lack a functional trehalose cycle and are unable to grow on glucose. A shift to high glucose leads to the accumulation of FBP with low ATP and P_i_ levels, suggesting an imbalance in glycolytic fluxes. Mechanisms ascribed to the trehalose pathway implied an involvement in the regulation of substrate demand by the upper- and lower-parts of glycolysis, either by (1) direct inhibition of hexokinase (hxk) - the first ATP consuming step - by an intermediate of this pathways, trehalose 6-phosphate (T6P), or (2) enhancement of glyceraldehyde 3-phosphate dehydrogenase (GAPDH) activity - the first reaction in lower glycolysis - through supply of P_i_. However, neither of these mechanisms could fully explain the metabolic deficits of *tps1*Δ mutants.

In this study, we started by exploring the consequences of a defective trehalose cycle in *S. cerevisiae* from a systems-level perspective. A detailed kinetic model of glycolysis allowed us to simulate the *tps1*Δ metabolic behavior. This model reproduced the most relevant metabolic features of the *tps1*Δ phenotype (ATP and P_i_ depletion, FBP accumulation and a low glycolytic flux). Surprisingly, we found that with the same parameter settings a second state, with normal glycolytic function, could be achieved under different initial conditions (e.g. higher P_i_ concentration). This bistable behavior of the model suggested the possibility that two phenotypes, viable and non-viable, could be present in the same population. We pursued this observation experimentally, reasoning that cells in the normal state should be viable. Using plating assays we found that, not only did such a small fraction of cells exist (~1 in 10^3^ cells), but it appeared spontaneously and reproducibly in any population of sufficient size. Propagation experiments showed that this glucose tolerance was non-genetic in nature. Additionally, this result provided an explanation for population level growth behaviors, which showed extremely long lag phases (> 20hrs). Further testing showed that such mixed population structures also explained the growth profiles and lag behaviors of *tps1*Δ populations grown on galactose with increasing amounts of glucose. In evaluating previous studies we found these results to be particularly informative in explaining population-level measurements for *tps1*Δ and related mutants.

Growth happens on much longer time scales than the metabolic processes for which we predicted the bistable behavior; could we also show distinct metabolic states at the single cell level? We exploited the fact that cells in the imbalanced state cannot maintain pH homeostasis, a consequence of low ATP levels, to identify the two states (low and normal pH) within a single population (Figure 1). For this we used a pH-dependent GFP reporter (pHluorin), combined with high throughput flow cytometry, and confirmed the presence of two distinct metabolic subpopulations in *tps1*Δ cultures. Significantly, our results revealed that a surprisingly large fraction of *wild-type *cells (7 %) also failed to properly initiate glycolysis.

**Figure 1:**
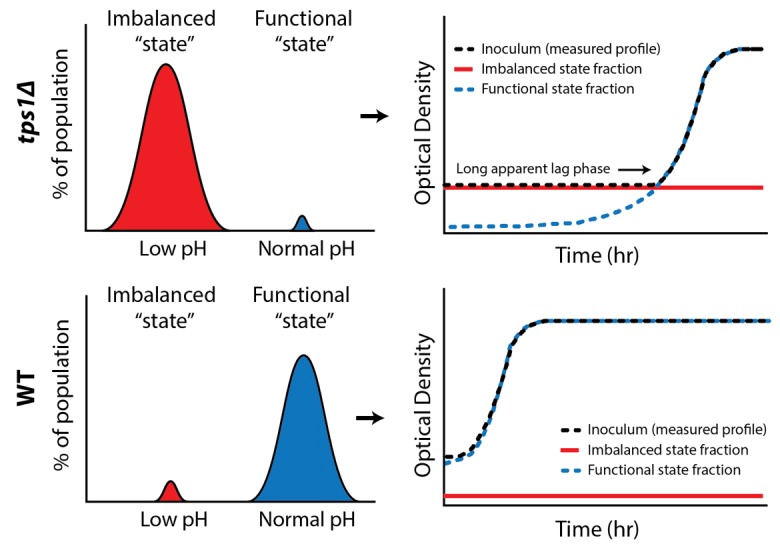
FIGURE 1: Bistability in glycolysis leads to two distinct subpopulations. Intracellular pH measurements reveal such metabolic subpopulations, with sizes that correspond to observed growth behaviors.

Next, we showed how this type of phenotypic heterogeneity could arise in metabolism through small continuously distributed variations in metabolic variables, rather than stochastic fluctuations. We generated > 10^6^ unique *tps1*Δ models, each with slightly varied initial metabolite concentrations and enzyme levels (V_max_); a situation likely for a population consisting of millions of individual cells. In response to a simulated glucose pulse, approximately 1 in 10^3^ models reached a functional steady state. Importantly, this approach allowed us to identify metabolic variables that could compensate for the absence of a trehalose pathway. We found that the successful initiation of glycolysis depended not so much on a single mechanism, but is determined by the interplay between components that tend to reduce the flux through the upper part of glycolysis (ATP consuming ) and those that enhance the flux through the lower (ATP producing) part. Further analysis showed that enhanced futile cycling of ATP would provide a consolidation of the identified mechanisms that together would achieve a balance between upper- and lower glycolytic activities (Figure 2).

**Figure 2:**
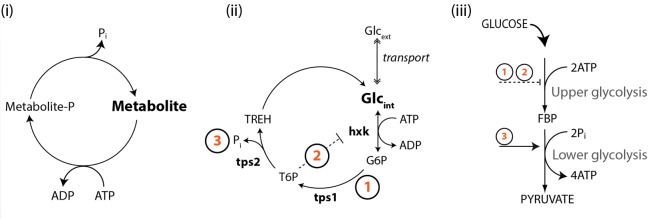
FIGURE 2: All the defining features of a futile cycle (i) are present in the full trehalose cycle (ii). Balancing upper- and lower-glycolytic fluxes during the initiation of glycolysis is achieved by a combination (iii) of features inherent in the trehalose cycle. The specific regulatory components include: (1) ATP hydrolysis and substrate removal and (2) T6P-mediated inhibition of hxk activity, which together slow down the upper-glycolytic flux, and (3) P_i_ liberation, which functions to stimulate lower glycolysis. Glc, glucose; G6P, glucose 6-phosphate; TREH, trehalose; tps2, trehalose 6-phosphate phosphatase.

We then realized that the full trehalose pathway constitutes a futile cycle, and that its temporary activation in response to glucose would be an effective way to balance the demand and supply of ATP with P_i_ availability. Estimations of *in vivo* fluxes, using a ^13^C-tracer approach, confirmed that this cycle is indeed transiently activated, with a highly dynamic response during shifts to high glucose. As much as 28% percent of the uptake flux was channeled toward trehalose, significantly impacting both the overall glycolytic flux and phosphate homeostasis. The flux profiles were fully consistent with our model’s prediction that various features inherent in this futile cycle (T6P mediated inhibition, ATP hydrolysis and P_i_ liberation and substrate removal) all contribute (Figure 2) to the robust startup of glycolysis. We could now also understand the seemingly incomplete mechanistic descriptions offered by previous interpretations.

A dynamic picture of regulation emerged: if glucose is suddenly available, its rapid phosphorylation in the upper part of glycolysis results in a drain on ATP and P_i_. Depending on the rate of glucose phosphorylation, the system may reach critically low P_i_ and ATP levels that could result in an imbalance between fluxes through the upper- and lower-parts of glycolysis. If, however, mechanisms kick in that slow down the upper part and/or P_i_ recovery is enhanced (i.e. stimulating the lower part), a balanced state can be reached. Importantly, this only made sense after the dynamics of the whole glycolytic pathway - its bistability - was considered.

While this study solved the long standing puzzle of the trehalose cycle’s involvement in glycolytic transitions, it was intriguing that 7 % of wild-type cells also failed to cope. Apparently the proper startup of glycolysis is not guaranteed even when the necessary regulatory components are available. How can this failure be understood? We speculate that the wild-type failure reflects a trade-off between efficient start-up and failsafe regulation. Although cells could reduce the startup risk by always maintaining the trehalose cycle in a high state of activity, effecting tighter control of glucose influx will come at a permanent cost of reduced ATP yield and flux capacity. From a population perspective a 7 % loss in viability could be justified if the remaining 93 % survives without a permanent sacrifice in capacity. Such a trade-off scenario has been shown, in many other contexts, to be an outcome of evolution in dynamic environments.

This work underscored the importance of specific mechanisms which dynamically regulate metabolism during environmental changes. It seems likely that such mechanisms are of general importance in natural environments, where changes can be transient and occur very suddenly without much forewarning. It may not be a coincidence that a pathway implied in heat and osmostress response is used by yeast also to cope with “glucose stress”.

